# Multiscale Structural Modulation and Synergistic Enhancement of Transparency and Relaxor Behavior in La^3+^-Doped KNN Lead-Free Ceramics

**DOI:** 10.3390/nano16020149

**Published:** 2026-01-22

**Authors:** Xu Yang, Lingzhi Wang, Li Luo, Wenjuan Wu, Bo Wu, Junjie Li, Jie Li, Tixian Zeng, Gengpei Xia

**Affiliations:** 1 Sichuan Province Key Laboratory of Information Materials and Devices Application, College of Optoelectronic Engineering (Chengdu IC Valley Industrial College), Chengdu University of Information Technology, Chengdu 610225, China; 2 Sichuan Meteorological Optoelectronic Sensor Technology and Application Engineering Research Center, Chengdu University of Information Technology, Chengdu 610225, China; 3 Sichuan Province Key Laboratory of Information Materials, Southwest Minzu University, Chengdu 610041, China; 4 Dazhou Industrial Technology Research Institute, Dazhou 635000, China; 5 CMA Key Laboratory of Cloud-Precipitation Physics and Weather Modification, Beijing 100081, China; 6 Chengdu Product Quality Inspection and Research Institute Co., Ltd., Chengdu 610199, China

**Keywords:** lead-free transparent ferroelectric ceramics, potassium sodium niobate, La^3+^ doping, nanoscale structural engineering, multifunctional properties

## Abstract

Lead-free transparent ferroelectric ceramics with integrated opto-electro-mechanical functionalities are pivotal for next-generation multifunctional devices. In this study, K_0.48_Na_0.52_NbO_3_-*x*La_2_O_3_ (KNN-*x*La, *x* = 0.005 − 0.04) ceramics were fabricated via a conventional solid-state route to investigate the La^3+^-induced multiscale structural evolution and its modulation of optical and electrical properties. La^3+^ substitution drives a critical structural transition from an anisotropic orthorhombic phase (*Amm*2) to a high-symmetry pseudocubic-like tetragonal phase (*P*4*mm*) for *x* ≥ 0.025, characterized by minimal lattice distortion (c/a = 1.0052). This enhanced structural isotropy, coupled with submicron grain refinement (<1 μm) driven by $V_{A}^{\prime }$-mediated solute drag, effectively suppresses light scattering. Consequently, a high-transparency plateau (*T*_780_ ≈ 53–58%, *T*_1700_ ≈ 70–72%) is achieved for 0.025 ≤ *x* ≤ 0.035. Simultaneously, the system undergoes a crossover from normal ferroelectric (FE) to relaxor (RF) state, governed by an FE–RF boundary at *x* = 0.015. While *x* = 0.005 exhibits robust piezoelectricity (*d*_33_ ≈ 92 pC/N), the *x* = 0.015 composition facilitates a transitional polar state with large strain (0.179%) and high polarization (*P*_m_ ≈ 33.3 μC/cm^2^, *P*_r_ ≈ 15.8 μC/cm^2^). Piezoresponse force microscopy (PFM) confirms the domain evolution from lamellar macro-domains to speckle-like polar nanoregions (PNRs), elucidating the intrinsic trade-off between optical transparency and piezoelectricity. This work underscores La^3+^ as a potent structural modifier for tailoring phase boundaries and defect chemistry, providing a cost-effective framework for developing high-performance transparent electromechanical materials.

## 1. Introduction

Transparent ferroelectric ceramics represent a novel class of advanced materials that synergize optical transparency with ferroelectric functionality. Their uniqueness originates from tailored crystal structures that enable opto-electro-mechanical coupling effects, demonstrating significant application potential in energy storage, intelligent sensing, and optoelectronic modulation [1,2]. Traditional lead-based systems, as exemplified by (Pb,La) (Zr,Ti)O_3_ (PLZT) ceramics, have long dominated the field primarily owing to their exceptional piezoelectric properties and significant electro-optic effects. However, environmental concerns arising from lead toxicity have driven a global research paradigm shift toward lead-free alternatives. Among these, perovskite-structured (K,Na)NbO_3_ (KNN)-based ceramics have emerged as particularly promising candidates due to their eco-friendliness and tunable functional properties. Current research progress and technical challenges can be systematically analyzed across three critical dimensions: material composition engineering, property optimization strategies, and fabrication process development [3,4,5].

Since the pioneering work on KNN-based relaxors via SrTiO_3_ incorporation in 2004 [6], extensive efforts have been dedicated to optimizing performance through multiscale structural engineering. The strategic integration of relaxor-type ABO_3_ additives, such as Sr(Sc_0.5_Nb_0.5_)O_3_ and Sr(Mg_1/3_Nb_2/3_)O_3_, has markedly improved optical transmittance across visible and infrared wavelengths [7,8,9]. Concurrently, oxide doping approaches involving La_2_O_3_ and SrZrO_3_ + Er_2_O_3_ composites have refined dielectric properties via grain boundary modification and defect engineering [10,11]. Crucially, recent insights highlight that defects in perovskite oxides are not merely accidental imperfections but can serve as active elements to strategically modulate electromechanical responses [12]. Specifically, defect dipoles can reconfigure the local energy landscape, thereby facilitating polarization rotation and enhancing functional properties. Building upon these advancements, the research scope has expanded from fundamental ferroelectricity and transparency to multifunctional integration, including piezoelectric effects, energy storage properties, and electro-optic coupling [13,14]. Despite these advancements, there remains an intrinsic conflict between phase structure and performance: high-symmetry pseudocubic phases improve transparency by minimizing birefringence, but often at the expense of ferroelectric activity, while high piezoelectricity typically relies on low-symmetry orthorhombic phases. Achieving synergistic optimization of optical transparency and electrical performance remains a critical scientific challenge.

Current emerging techniques for fabricating KNN-based transparent ceramics, including hot pressing, controlled atmosphere sintering, and spark plasma sintering (SPS), face critical limitations such as complex processing requirements and high equipment costs. For instance, while hot pressing effectively reduces porosity, it demands precise control of pressure and temperature gradients. Similarly, although SPS enables rapid densification, it struggles to achieve uniform grain size distribution [15,16,17]. These technical bottlenecks significantly hinder the large-scale industrial adoption of transparent ceramic materials, making the development of cost-effective and scalable fabrication methods a pressing technical challenge in the field.

To address these challenges, this study establishes a rigorous scientific hypothesis based on defect-mediated multiscale structure tailoring. We selected the composition K_0.48_Na_0.52_NbO_3_ as the base material due to its proximity to the polymorphic phase boundary, which offers a robust intrinsic piezoelectric response to buffer the performance trade-offs required for transparency. We postulate that La^3+^ donor doping functions as a “multi-scale structural modifier” via a defect-driven mechanism: (1) At the atomic scale, La^3+^ substitution generates A-site cation vacancies ($V_{A}^{\prime }$) to maintain charge neutrality; (2) At the mesoscale, these charged defects and resulting random fields disrupt long-range ferroelectric order, driving the transition from light-scattering macro-domains to optically isotropic polar nanoregions (PNRs); (3) At the microscale, defect-mediated grain boundary pinning effectively inhibits grain growth. Concurrently, the traditional solid-state reaction method is systematically refined to achieve high densification without high-pressure assistance. A comprehensive investigation focuses on elucidating the defect-induced phase evolution mechanisms in the K_0.48_Na_0.52_NbO_3_-*x*La_2_O_3_ system, with particular emphasis on structure-property correlations involving microstructural characteristics, optical performance (e.g., transmittance), and electrical functionalities (e.g., dielectric, ferroelectric, and piezoelectric responses). This work aims to overcome the performance trade-off between transparency and functional properties while establishing a material foundation for developing multifunctional integrated devices.

## 2. Experimental Section

### 2.1. Material Preparation

K_0.48_Na_0.52_NbO_3_-*x*La_2_O_3_ (KNN-*x*La, *x* = 0.005 − 0.04 mol) ceramics were synthesized via a conventional solid-state reaction method. The base composition with a K/Na ratio of 0.48/0.52 was selected due to its proximity to the polymorphic phase boundary (PPB), providing a robust ferroelectric baseline for subsequent domain manipulation. High-purity raw materials—K_2_CO_3_ (99.0%), Na_2_CO_3_ (99.8%), Nb_2_O_5_ (99.5%), and La_2_O_3_ (99.9%) (all from Sinopharm Chemical Reagent Co., Ltd., Shanghai, China)—were precisely weighed according to stoichiometric proportions. The powder mixtures underwent planetary ball milling (400 rpm, 10 h) in anhydrous ethanol using ZrO_2_ media. After drying, the homogenized powders were calcined at 850 °C for 5 h, followed by re-milling (8 h) to ensure particle uniformity. Granulation was performed using a 5–7 wt% polyvinyl alcohol (PVA) aqueous binder, after which the powders were uniaxially pressed into disk-shaped compacts (Φ10 mm × 1 mm) under 8 MPa. Binder burnout was achieved through thermal decomposition at 500–850 °C for 5 h. Final sintering occurred at 1180 °C for 3–5 h in a powder-bed environment (using compensatory powders of the same composition) to suppress the volatilization of alkali elements (K/Na). For optical characterization, the sintered pellets were polished to an optical-grade mirror finish (final thickness of 0.4 mm) using a multi-stage protocol with SiC papers (up to 7000 mesh) and diamond suspensions (down to 0.5 μm). Silver electrodes were screen-printed on both surfaces and fired at 600 °C for 10 min to establish electrical contact.

### 2.2. Material Characterization

The phase composition was analyzed via X-ray diffraction (XRD-6100, Shimadzu Corporation, Kyoto, Japan) with Cu-Kα radiation (*λ* = 1.5406 Å). Detailed structural evolution and lattice parameters were determined through full-pattern Rietveld refinement using the GSAS-II software package (version [5.6.0], Argonne National Laboratory, Argonne, IL, USA). Microstructural morphology and elemental distributions were characterized using scanning electron microscopy (SEM) equipped with energy-dispersive X-ray spectroscopy (EDS) (Phenom XL, Thermo Fisher Scientific Inc., Waltham, MA, USA). Chemical states and defect chemistry were investigated via X-ray photoelectron spectroscopy (XPS, K-Alpha, Thermo Fisher Scientific Inc., Waltham, MA, USA) with all binding energies calibrated to the adventitious C 1s peak (284.8 eV). Temperature-dependent dielectric properties were measured on unpoled samples from 30 °C to 450 °C during the heating process at a constant rate of 2 °C/min, using an impedance analyzer (WK6500P, Wayne Kerr Electronic Instrument Co., London, UK) coupled with a high-low temperature system (DMS-2000, Partulab Technology Co., Ltd., Wuhan, China) over a frequency range of 100 Hz to 1 MHz. Ferroelectric and strain properties were evaluated at 1 Hz using a precision ferroelectric test system (TF Analyzer 2000 E, aix-ACCT Systems GmbH, Aachen, Germany) with a 10 kV high-voltage amplifier. A triangular waveform was applied for two consecutive cycles, and data from the second cycle adopted for analysis to ensure domain switching stability. Piezoelectric coefficient (*d*_33_) was measured using a quasi-static *d*_33_ m (ZJ-3A, Institute of Acoustics, Chinese Academy of Sciences, Beijing, China) after poling the samples in a 60 °C silicone oil bath. Domain structures were characterized by Vertical Piezoresponse Force Microscopy (VPFM, MFP-3D, Oxford Instruments Asylum Research Inc., Santa Barbara, CA, USA) in contact mode with an AC excitation bias of 2 V.

## 3. Results and Discussion

### 3.1. Optical Properties

**Figure 1:**
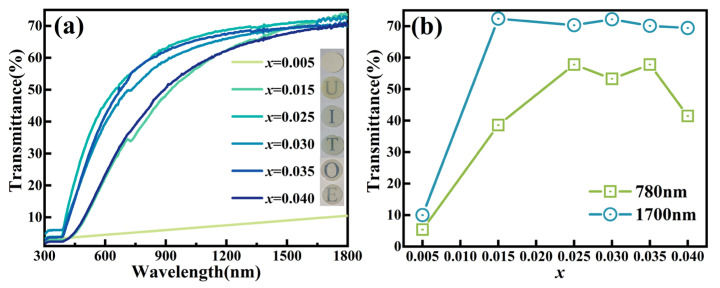
Optical transmittance of KNN-*x*La ceramics: (**a**) 300–1800 nm (Inset: photographs demonstrating transparency), (**b**) transmittance at 780 nm and 1700 nm as a function of *x*.

Figure 1a presents the optical transmittance spectra of unpoled KNN-*x*La ceramics in the wavelength range of 300–1800 nm, with inset photographs illustrating mirror-polished specimens (0.4 mm in thickness). All compositions exhibit near-zero transmittance in the 300–400 nm ultraviolet range, which is attributable to the fundamental absorption edge [18,19]. Notably, the low-doped ceramic (*x* = 0.005) demonstrates poor transparency (transmittance <10%), whereas higher La^3+^ concentrations (*x* ≥ 0.015) significantly enhance transparency. Specifically, the near-infrared transmittance (*λ* = 1700 nm) reaches a maximum of 72.2% at *x* = 0.030. As shown in Figure 1b, the visible-range transmittance (*λ* = 780 nm) displays a non-monotonic doping dependence: the transparency first increases, reaching a peak at *x* = 0.035 (*T*_780 nm_ = 57.8%), and then slightly degrades at *x* = 0.04. The *x* = 0.005 sample appears opaque (text illegibility), while progressive La^3+^ incorporation improves transparency until excessive doping (*x* = 0.04) induces light scattering losses. It is noteworthy that a high-transparency plateau (*T*_780 nm_ ~ 53–58%, *T*_1700 nm_ ~ 70–72%) is maintained within the composition range of 0.025 ≤ *x* ≤ 0.035. This optimal performance represents a highly competitive level among KNN-based ceramics prepared via conventional sintering [20,21,22,23,24,25], confirming that La^3+^ doping acts as an effective multiscale structural modifier to favorably balance optical and functional properties.

**Figure 2:**
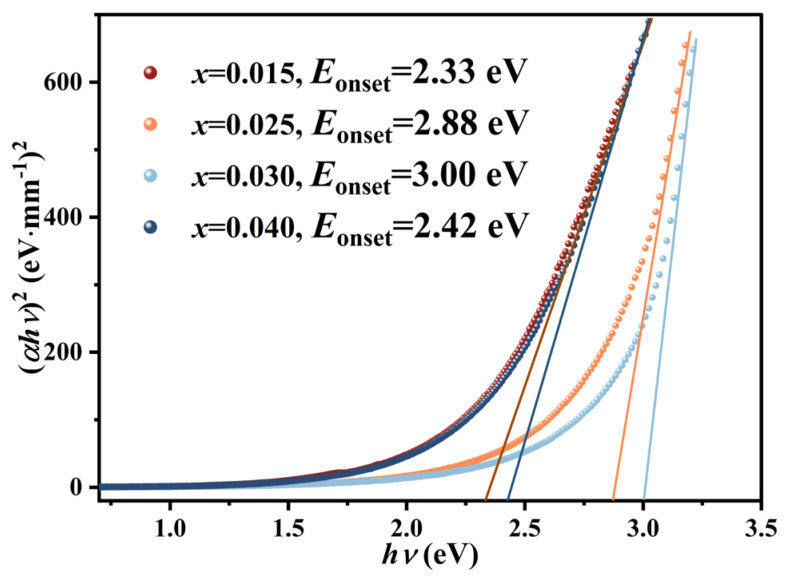
The plot of (*hνα*)^2^ versus *hν* and the extracted optical absorption onset (*E*_onset_) for KNN-*x*La ceramics.

Figure 2 illustrates the systematic modulation of the optical absorption onset (*E*_onset_) in KNN-*x*La ceramics (*x* ≥ 0.015). To ensure a rigorous interpretation of the spectroscopic data [26], the term *E*_onset_ is adopted instead of “fundamental bandgap” to more accurately reflect the absorption edge, which may be influenced by defect states. The values were determined via the Tauc relation for direct electronic transitions:

(*hνα*)^2^ = *A*(*hν − E*_conset_) (1)

where α = 1/*d* ln(1/*T*) is the absorption coefficient (with *d* = 0.4 mm). As shown in Figure 2, *E*_onset_ initially blue-shifts from 2.33 eV (*x* = 0.015) to a maximum of 3.00 eV (*x* = 0.030), before red-shifting at higher doping levels. The initial expansion of *E*_onset_ stems from the lattice distortion and electronic band structure modulation induced by ${\mathrm{La}}_{Ä}$ substitution, which effectively suppresses parasitic interband transitions in the short-wavelength region. However, the subsequent reduction in *E*_onset_ at *x* = 0.040 is closely linked to the proliferation of doping-induced complex defects (e.g., cation vacancies $V_{A}^{\prime }$) and the associated Urbach tails in the density of states [27]. These defect-induced sub-bandgap states act as localized absorption centers, shifting the absorption edge toward lower energies. Consequently, the composition at *x* = 0.030 achieves the maximum *E*_onset_ (3.00 eV), representing the optimal suppression of defect-mediated absorption. These results underscore that defect-engineered band-edge modulation is a critical mechanism for enhancing the photonic response in KNN-based transparent ferroelectrics.

### 3.2. Phase Structure and Microstructure

**Figure 3:**
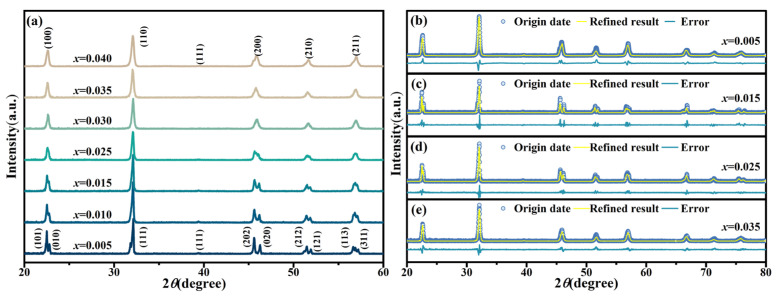
(**a**) X-ray diffraction (XRD) patterns and (**b**–**e**) Rietveld refinement of KNN-*x*La ceramics.

Figure 3a displays the room-temperature XRD patterns of KNN-*x*La ceramics. All samples exhibit a pure perovskite structure without detectable secondary phases, indicating that La^3+^ has been completely incorporated into the KNN lattice to form a stable solid solution. To provide a quantitative understanding of the doping-induced structural evolution, full-pattern Rietveld refinements were performed (Figure 3b–e), with the refined lattice parameters, weight fractions of phases, and refinement reliability factors (*R*_wp_) summarized in Table 1. At low concentrations (*x* ≤ 0.015), the ceramics are dominated by an orthorhombic (O) phase (*Amm*2), with weight fraction of 86.19% for *x* = 0.005 and 97.37% for *x* = 0.015. The distinct splitting of (101)/(010) and (002)/(200) peaks confirm the high degree of lattice anisotropy characteristic of the O-phase. A critical structural transition occurs at *x* ≥ 0.025, where the symmetry evolves into a predominant tetragonal (T) phase (*P*4*mm*).

Quantitatively, Table 1 reveals that for the T phase, the c/a ratio decreases from 1.0072 (*x* = 0.015) to a minimum of 1.0052 (*x* = 0.025) and remains at a low level through *x* = 0.035. The extremely low lattice distortion, approaching that of cubic phases, indicates a pseudocubic-like configuration. This reduction in lattice distortion—driven by the compressive strain induced by substituting host K^+^/Na^+^ ions with the smaller La^3+^ (1.36 Å, CN = 12)—is the fundamental origin of the enhanced transparency. A lower c/a ratio signifies an approach toward optical isotropy, which effectively minimizes the refractive index mismatch across grain boundaries and reduces birefringence-induced light scattering. Consequently, the compositions within the 0.025 ≤ *x* ≤ 0.035 range achieve a synergistic optimization of high symmetry and minimal lattice anisotropy, thereby sustaining the optimal optical isotropy and the resultant transmittance plateau.

**Figure 4:**
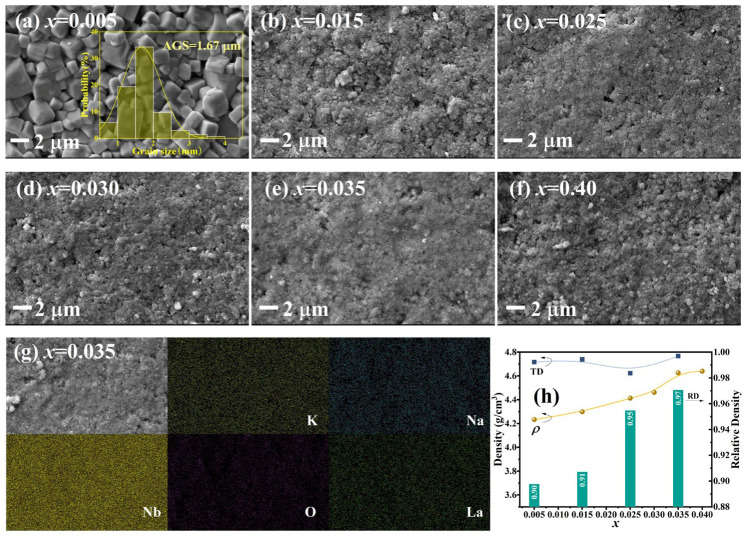
(**a**–**f**) Surface SEM (Inset is the grain size distribution), (**g**) elemental mappings, (**h**) densities (*ρ*), theory densities (TD), and relative densities (RD) for KNN-*x*La ceramics (The arrows indicate the corresponding Y-axes (left or right) for the respective data series.).

Figure 4a–f illustrate the surface morphologies of KNN-*x*La ceramics. The *x* = 0.005 sample displays well-defined grains with a clear tetragonal-like morphology and an average grain size (AGS) of 1.67 μm. Increasing La^3+^ concentration triggers significant grain refinement, reducing the AGS to submicron levels (<1 μm) for *x* ≥ 0.015. This grain growth inhibition is governed by the “solute drag” effect and defect-mediated pinning: the substitution of La^3+^ at A-sites generates cation vacancies ($V_{A}^{\prime }$) for charge compensation. These vacancies accumulate at grain boundaries, pining their migration during sintering [13,27,28]. To verify the chemical homogeneity, EDS mapping for the *x* = 0.035 sample was performed (Figure 4g). The uniform distribution of K, Na, Nb, O, and especially La across the grain and boundary regions confirms that La^3+^ has been successfully incorporated into the perovskite lattice without forming La-rich secondary phases or segregations or elemental segregation.

The bulk experimental density (*ρ*) of KNN-*x*La ceramics was measured using the Archimedes method. The theoretical density (TD) was calculated from the unit cell parameters obtained via Rietveld refinement of the XRD patterns, allowing the relative density (RD) to be determined by RD = (*ρ*/TD) × 100%. The evolution of *ρ*, TD, and RD as a function of *x* is summarized in Figure 4h. The RD increases steadily with La content, exceeding 95% for *x* ≥ 0.025 and reaching over 97% for the *x* = 0.035 composition. This high densification, combined with the observed submicron grain size, effectively minimizes pore-induced light scattering. Synergistically, the transition to a high-symmetry pseudocubic-like tetragonal phase (as discussed in Figure 3) and the suppression of grain-boundary scattering via microstructural refinement collectively contribute to the outstanding transparency (*T*_780 nm_ ≈ 53–58%, *T*_1700 nm_ ≈ 70–72%) sustained within the 0.025 ≤ *x* ≤ 0.035 range.

### 3.3. XPS Analysis and Defect Chemistry

**Figure 5:**
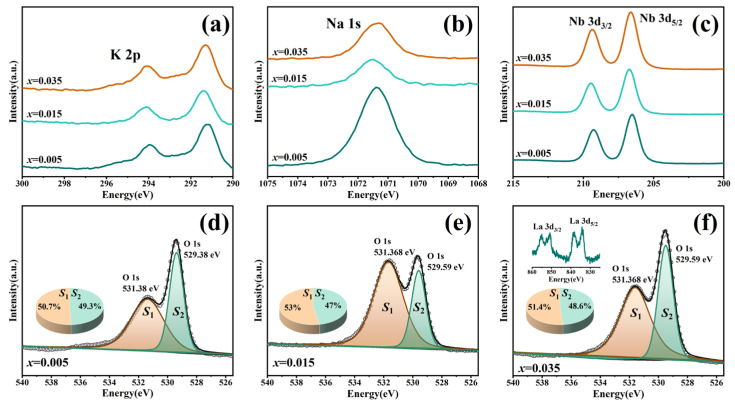
XPS spectra of KNN-*x*La ceramics with *x* = 0.005, 0.015, and 0.035: (**a**) K 2*p*, (**b**) Na 1*s*, (**c**) Nb 3*d*, and (**d**–**f**) deconvoluted O 1*s* (oxygen vacancy in different components). The O 1*s* peaks are fitted into two components: *S*1 (oxygen-vacancy-related species, O_vac_) and *S*2 (lattice oxygen, O_lat_).

To verify the chemical state of the dopants and establish a rigorous defect-chemistry framework, high-resolution XPS spectra of La 3d, K 2*p*, Na 1*s*, Nb 3*d* and O 1*s* were acquired, as illustrated in Figure 5. As shown in the inset of Figure 5f, the La 3*d* core-level spectrum exhibits characteristic multiplet splitting, with the 3*d*_5/2_ and 3*d*_3/2_ peaks positioned at approximately 834.7 eV and 851.5 eV, respectively. These binding energies (BEs), along with the distinct satellite peaks, confirm that La exists in the +3 oxidation state and has been successfully incorporated into the perovskite lattice [6,25]. The K 2*p*, Na 1*s*, and Nb 3*d* spectra (Figure 5a–c) exhibit measurable BE shifts with varying *x*, though the peak profiles indicate that La^3+^ substitution does not alter the fundamental valence states of the host cations. Specifically, the Nb 3*d* doublet (Nb 3*d*_5/2_ ≈ 206.5 eV and Nb 3*d*_3/2_ ≈ 209.3 eV) corresponds strictly to the Nb^5+^ state. The conspicuous absence of Nb^4+^ signals (≈204.0 eV) is a critical finding, as it definitively rules out electronic compensation (Nb^5+^ → Nb^4+^) as a primary mechanism for charge balance.

Given the A-site substitution by the aliovalent La^3+^ donor, the charge-neutrality must consequently be maintained through an ionic compensation mechanism. In donor-doped alkali niobates, this is primarily achieved via the creation of cation vacancies ($V_{A}^{\prime }$) to balance the excess positive charge of ${\mathrm{La}}_{Ä}$ sites. The corresponding Kröger–Vink equation is expressed as:

(2) $$La_{2}O_{3}\overset{KNN}{\to }2{\mathrm{La}}_{Ä}+2V_{A}^{\prime }+3O_{O}^{\times }$$

These $V_{A}^{\prime }$ defects are instrumental in the observed grain refinement (Figure 4), as they segregate at grain boundaries and exert a solute drag effect on boundary migration.

To further quantify the impact on the oxygen sublattice, the O 1*s* spectra were deconvoluted into lattice oxygen (O_lat_ ≈ 529.5 eV) and oxygen-vacancy-related species (O_vac_ ≈ 531.4 eV) (Figure 5d–f). The relative concentration of O_vac_ (S_1_) exhibits a non-monotonic evolution. For *x* = 0.005, the O_vac_ ratio is 50.7%, which increases to a maximum of 53.0% at *x* = 0.015—coinciding with the period of peak structural instability near the phase boundary. However, at *x* = 0.035, the O_vac_ ratio is suppressed to 51.4%. This suggests that at high doping levels, the A-site vacancy compensation mechanism ($V_{A}^{\prime }$) becomes dominant, effectively stabilizing the lattice and inhibiting the proliferation of intrinsic oxygen vacancies. This shift in defect dominance from $V_{Ö}$ to $V_{A}^{\prime }$ is expected to significantly reduce leakage current and enhance the optical response of the KNN-*x*La ceramics.

### 3.4. Dielectric Properties

**Figure 6:**
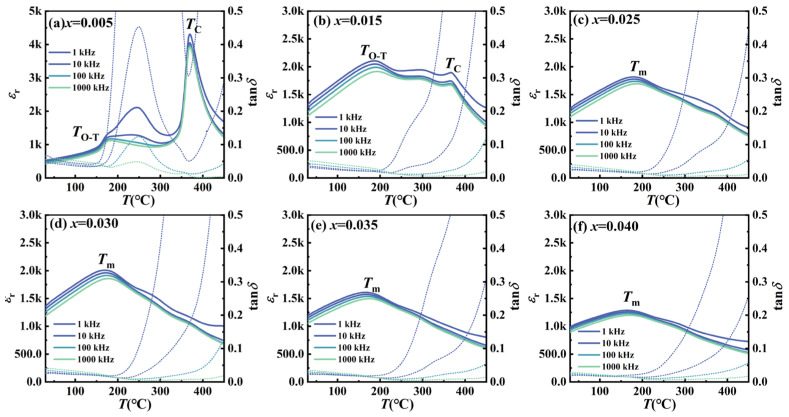
Temperature dependence of dielectric constants (*ε*_r_) and dielectric loss (tan*δ*) for KNN-*x*La ceramics. Solid and dotted lines represent *ε*_r_ and tan*δ*, respectively.

Figure 6 illustrates the temperature-dependent dielectric constant (*ε*_r_) and dielectric loss (tan*δ*) of unpoled KNN-*x*La ceramics measured from 30 °C to 450 °C within the frequency range of 1 kHz − 1 MHz. For *x* = 0.005, the *ε*_r_-*T* profile displays two distinct dielectric anomalies characteristic of pristine KNN: the orthorhombic-to-tetragonal phase transition (*T*_O−T_ ≈ 170 °C) and the tetragonal-to-cubic (*T*_C_ ≈ 370 °C) transitions. Notably, a broad anomalous dielectric peak emerges between 200 °C and 300 °C. Following the defect-chemistry framework discussed in the XPS section, this anomaly is ascribed to the thermally activated hopping of defect dipoles (e.g., ${\mathrm{La}}_{Ä}-V_{A}^{\prime }$ complexes) formed via donor-doping-induced charge compensation.

Combined with the Rietveld refinement results (Table 1), the *x* = 0.015 composition maintains the *T*_O−T_ peak due to the continued dominance of the O (*Amm*2) phase (97.37 wt%). However, a profound dielectric crossover from normal ferroelectric to a relaxor behavior occurs at *x* ≥ 0.025. The discrete transitions merge into a single, broad dielectric anomaly (*T*_m_ ≈ 160 °C) with significant frequency dispersion (i.e., *T*_m_ shifts toward higher temperatures with frequency), a hallmark of relaxor ferroelectrics. This diffusion correlates with the stabilization of the high symmetry pseudocubic-like tetragonal phase and the minimization of lattice distortion (*c*/*a* = 1.0052). Such structural evolution disrupts long-range ferroelectric order, promoting the formation of dynamic polar nanoregions (PNRs) that dominate the dielectric response.

**Figure 7:**
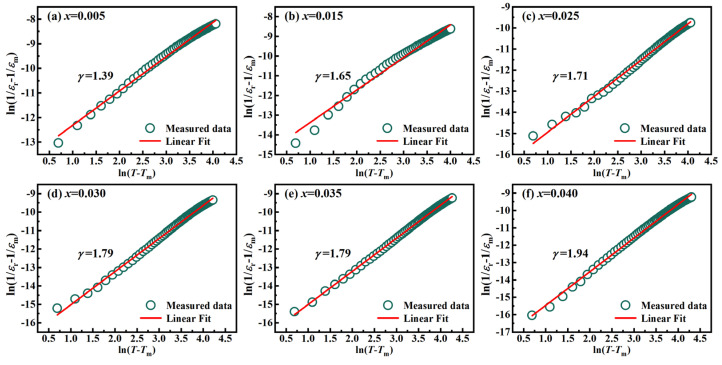
The plots of ln(*T* − *T*_m_) versus ln(1/*ε*_r_ − 1/*ε*_m_) of KNN-*x*La ceramics at 1 kHz.

To quantify this diffusivity, the modified Curie–Weiss law as follows is employed:

(3) $$\frac{1}{{ɛ}_{r}}-\frac{1}{{ɛ}_{m}}=\frac{{\left(T-T_{m}\right)}^{\gamma }}{C}$$

where *C* is the Curie-like constant and *γ* represents the degree of diffuseness (with *γ* = 1 for a normal ferroelectric and *γ* = 2 for an ideal relaxor). The *γ* value is determined from the slope of the ln(1/ɛ_r_ − 1/ɛ_m_) versus ln(*T* − *T*_m_) plots at 1 kHz (Figure 7), with the results summarized in Table 2. The *γ* value systematically increases from 1.39 (*x* = 0.005) to 1.94 (*x* = 0.040), indicating a robust transition from a near-normal ferroelectric to a distinct relaxor state.

The physical origin of this enhanced relaxation and structural evolution stems from La^3+^-mediated compositional disorder. The heterovalent substitution of K^+^/Na^+^ by La^3+^ induces local charge imbalances and lattice distortions, thereby generating random electric fields that disrupt long-range ferroelectric order. These random fields facilitate the fragmentation of macroscopic domains into dynamic polar nanoregions (PNRs). The enhanced athermal and thermal fluctuations of these PNRs not only drive the increase in *γ* but also explain the systematic suppression of peak *ε*_r_. Additionally, the submicron grain refinement (*x* ≥ 0.025) increases the density of non-polar grain boundaries, further broadening the transition profile. This transition to a high-symmetry, relaxation-dominated state is the fundamental origin of the optical isotropy and suppressed scattering, which collectively facilitate the superior transparency observed in the *x* ≥ 0.025 composition.

### 3.5. Ferroelectricity and Strain Properties

**Figure 8:**
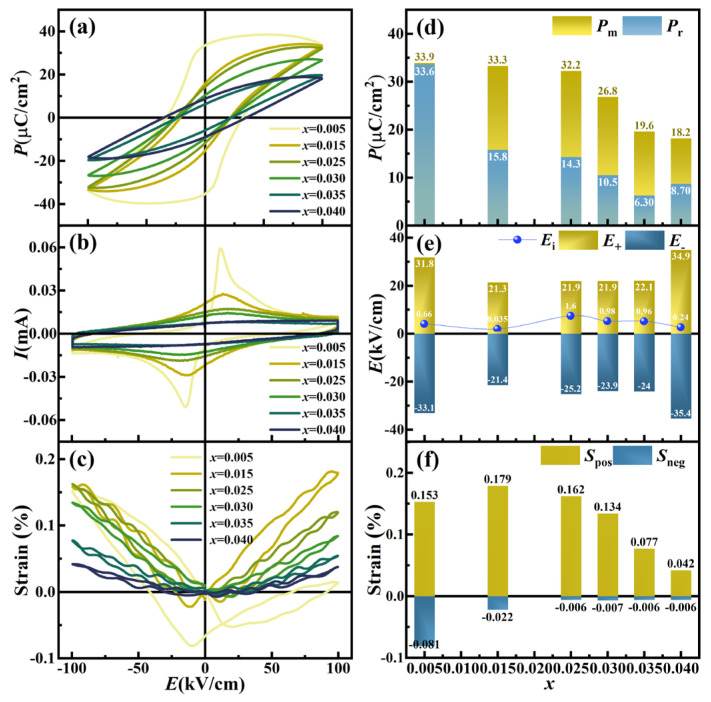
(**a**) *P*-*E* loops, (**b**) *I*-*E* curves, (**c**) *S*-*E* curves, (**d**) *P*_max_, *P*_r_, (**e**) *E*_+_, *E*_−_, *E*_i_, and (**f**) *S*_pos_, *S*_neg_ of KNN-*x*La ceramics.

**Figure 9:**
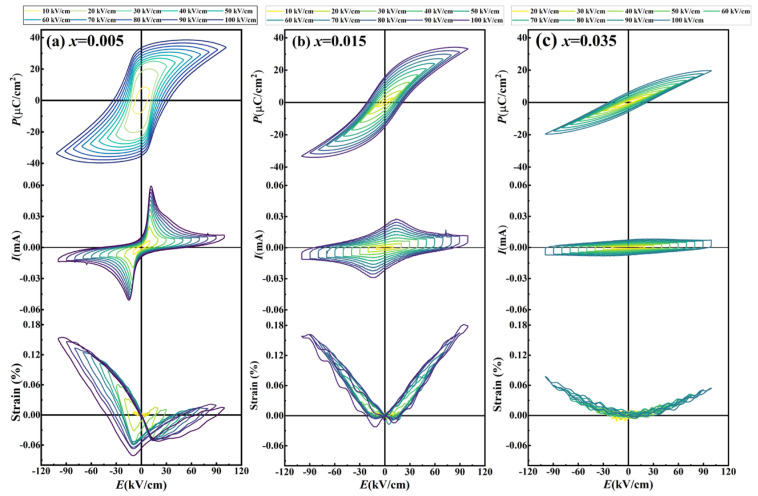
*P*-*E* loops, *I*-*E* curves, and *S*-*E* curves at various electric fields (*E*) for KNN-*x*La ceramics with (**a**) *x* = 0.005, (**b**) *x* = 0.015, and (**c**) *x* = 0.035.

Figure 8a,b systematically investigate the composition-dependent ferroelectricity of KNN-*x*La ceramics through polarization-electric field (*P*-*E*) hysteresis loops and current-electric field (*I*-*E*) curves measured at 1 Hz under 100 kV/cm. The polarization intensity (maximum polarization *P*_m_, remnant polarization *P*_r_), coercive electric field (positive electric field *E*_+_, negative electric field *E*_−_), and internal bias field (*E*_i_, calculated via *E*_i_ = (|*E*_+_| − |*E*_−_|)/2) as a function of doping content are shown in Figure 8d,e. The ceramics with *x* = 0.005 exhibit a near-rectangular *P*-*E* loop and sharp *I*-*E* switching peaks, confirming strong ferroelectricity (*P*_r_ ≈ 33.6 μC/cm^2^), which is a macroscopic manifestation of the long-range ferroelectric order of the orthorhombic (*Amm*2) phase identified in XRD (Figure 3). This composition maintains discrete *T*_O−T_ and *T*_C_ dielectric anomalies, highlighting its near-normal ferroelectric nature. The observed internal bias field (*E*_i_ ≈ 0.66 kV/cm) and significant leakage current are physically rooted in the ${\mathrm{La}}_{Ä}\;-\;V_{A}^{\prime }$ defect complexes. These defect dipoles, localized within the orthorhombic lattice, provide a stabilizing torque that pins the macroscopic domain walls. The *P*-*E*, *I*-*E*, and *S*-*E* curves for KNN-*x*La ceramics with *x* = 0.005 under varying electric fields (*E*) are provided in Figure 9. The *E*_i_ progressively strengthens with increasing applied electric field, amplifying the asymmetry in both *P*-*E* and *S*-*E* responses—a macroscopic manifestation of the synergistic effects of space charge and defect-dipole-mediated domain pinning.

As the La concentration increases to *x* = 0.015, the emergence of relaxor behavior induces a marked slimming of the *P*-*E* loops, characterized by a 52% reduction in *P*_r_ (15.8 μC/cm^2^) while maintaining a high *P*_m_ (33.3 μC/cm^2^). This behavior is rooted in the fragmentation of macroscopic domains into ergodic polar nanoregions (PNRs). Although Rietveld refinement (Table 1) shows a high content of orthorhombic phase (97.4%), the chemical disorder and trace cubic phase (0.2%) disrupt the cooperative long-range coupling, facilitating thermal randomization of PNRs upon field removal. This is further evidenced by the near-total dissipation of *E*_i_ (0.035 kV/cm), indicating that at this composition, thermal fluctuations of PNRs dominate over the pinning effects of defect dipoles.

For *x* ≥ 0.025, the system transitions fully into an ergodic relaxor state stabilized by the dominant pseudocubic-like tetragonal phase, as confirmed by the merging of split XRD peaks and relaxor-like dielectric anomalies (Figure 5 and Figure 6). Here, long-range order is entirely disrupted, yielding relaxor-typified *P*–*E* loops with significantly diminished *P*_m_ (19.6 μC/cm^2^ at *x* = 0.035). This suppression directly corresponds to the stabilization of the high-symmetry *P*4*mm* phase with minimal lattice distortion (c/a = 1.0052), which prevents the formation of long-range correlated spontaneous polarization. The enhanced compositional disorder inherent in this high-symmetry matrix stabilizes dynamic PNRs that randomly orient upon field removal, eliminating remanent polarization and yielding paraelectric-like loops. The transient enhancement of *E*_i_ (0.96–1.6 kV/cm) observed in these compositions may be attributed to local polarization gradients at the PNR-matrix interfaces, which create short-range electrostatic pinning under high-field excitation.

The strain-electric field (*S*-*E*) responses (Figure 8c) further validate this evolution. At *x* = 0.005, the typical butterfly curve exhibits significant asymmetry (*S*_pos_ ~ 0.153%, *S*_neg_ ~ 0.081%), a characteristic of non−180° domain wall motion facilitated by the O-T phase coexistence (*Amm*2 *+ P*4*mm*) and constrained by defect-induced pinning [29]. Interestingly, *x* = 0.015 marks the peak of positive strain (*S*_pos_ ~ 0.179%) while *S*_neg_ drops sharply (0.022%). This peak is ascribed to the highly reversible switching of PNRs within the *Amm*2 matrix, which provides a larger effective volume change than pure macroscopic domain switching. For *x* ≥ 0.025, the *S*-*E* curves transition to a quasi-linear, hysteresis-free shape with negligible *S*_neg_. This signifies a fundamental shift to electrostrictive behavior driven by lattice anharmonicity in the high-symmetry pseudocubic-like tetragonal *P*4*mm* phases. This low-hysteresis strain response is particularly advantageous for high-precision actuator applications, where it compensates for the loss of traditional piezoelectricity [30].

### 3.6. Piezoelectric Performance

**Figure 10:**
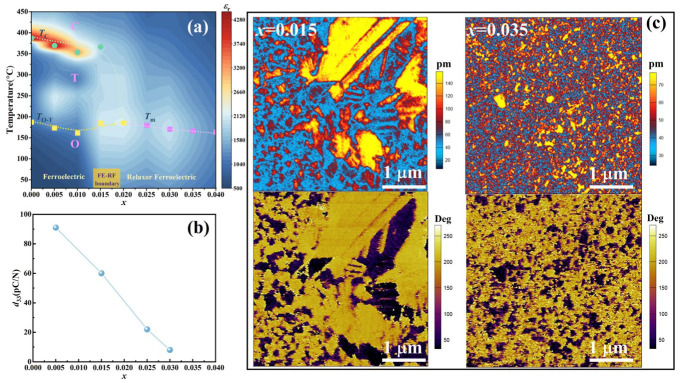
(**a**) Phase diagram, (**b**) *d*_33_, and (**c**) the vertical PFM images of KNN-*x*La ceramics.

The composition-temperature phase diagram for KNN-*x*La ceramics, constructed from dielectric temperature spectroscopy, is presented in Figure 10a. With increasing *x*, the Curie temperature (*T*_C_) decreases and merges with the orthorhombic-tetragonal transition temperature (*T*_O−T_), establishing a ferroelectric–relaxor (FE–RF) boundary at *x* = 0.015. For *x* ≥ 0.025, the convergence of these transitions, coupled with the stabilization of the high-symmetry pseudocubic-like tetragonal P4mm phase (Table 1), forms an orthorhombic–pseudocubic (O–PC) boundary. These two critical boundaries dictate the piezoelectric response shown in Figure 10b.

The piezoelectric coefficient (*d*_33_) exhibits a monotonous decline from 92 pC/N (*x* = 0.005) to 60 pC/N (*x* = 0.015), and eventually diminishes (<20 pC/N) for *x* ≥ 0.025. At *x* = 0.005, the robust piezoelectricity originates from a well-defined long-range ferroelectric order and strong spontaneous polarization of the orthorhombic lattice. Upon crossing the FE–RF boundary (*x* = 0.015), the reduction in *d*_33_ is ascribed to the fragmentation of macroscopic domains by nascent polar nanoregions (PNRs), which complicates the long-range polar coupling despite the high orthorhombic phase content. For *x* ≥ 0.025, the dominance of the pseudocubic-like P4mm phase eliminates the macroscopic polarization order, causing the dissipation of piezoelectric activity.

To visualize the microscopic domain evolution, piezoresponse force microscopy (PFM) was performed on *x* = 0.015 and *x* = 0.035 (Figure 10c). The *x* = 0.015 sample exhibits a multi-scale domain hierarchy where macro-scale lamellar (stripe) domains coexist with fine micro-domains. This configuration indicates a transitional polar state, where the material retains sufficient ferroelectric character for a moderate *d*_33_ while exhibiting significant relaxor-like strain enhancement [31]. In contrast, the *x* = 0.035 sample displays a “speckle-like” morphology dominated by PNRs with no traceable macroscopic domain walls. This confirms the complete transition to a relaxor state within an optically isotropic matrix. This structural evolution elucidates the fundamental mechanism for achieving record transmittance in heavily doped compositions at the expense of traditional piezoelectricity, underscoring the role of phase boundary engineering in tailoring multifunctional synergistic responses.

Table 3 provides a comprehensive comparison of the optical and piezoelectric performance between this work and other state-of-the-art KNN-based transparent systems. Utilizing a conventional solid-state sintering route, KNN-*x*La ceramics achieve a competitive balance, featuring a visible-light transmittance of ∼57% (∼72% in NIR) alongside a respectable *d*_33_ of 92 pC/N. Unlike many high-performance systems that require complex processing such as hot-pressing [17], our system offers a cost-effective pathway to multifunctional integration. This synergy is fundamentally rooted in the precise modulation of phase boundaries and domain architectures—specifically the transition from long-range ferroelectric order to a relaxation-dominated state—demonstrating that KNN-*x*La is a promising platform for next-generation transparent electromechanical devices.

## 4. Conclusions

In summary, KNN-*x*La transparent ceramics were successfully synthesized, demonstrating that La^3+^ doping serves as an effective multiscale structural lever to balance optical and functional properties. Structural analysis reveals that La^3+^ substitution at the A-site drives a transition from an orthorhombic phase to a high-symmetry pseudocubic-like tetragonal phase with minimal lattice distortion (c/a ≈ 1.0052), which provides the fundamental prerequisite for optical isotropy. Synergistically, the creation of cation vacancies ($V_{A}^{\prime }$) induces significant grain refinement (<1 μm) and high densification (>97%), suppressing grain-boundary scattering and sustaining a high-transparency plateau (*T*_780_ ≈ 53–58%, *T*_1700_ ≈ 70–72%) within the 0.025 ≤ x ≤ 0.035 range. Defect chemistry analysis via XPS confirms an ionic compensation mechanism ($V_{A}^{\prime }$) that stabilizes the lattice and modulates the optical absorption onset (*E*_onset_ ≈ 3.00 eV). Electrically, the ceramics evolve from a long-range ferroelectric order to an ergodic relaxor state. The composition at *x* = 0.015 marks a critical FE–RF boundary, achieving peak strain (0.179%) through the reversible switching of PNRs. These results clarify the structure–property correlations governed by phase boundary engineering and domain fragmentation, establishing KNN-*x*La as a competitive lead-free platform for integrated optoelectronic and electromechanical applications.

## Figures and Tables

**Table 1:** Results of Rietveld refinements for KNN-*x*La ceramics.

Parameters	Rwp (%)	Space Group (wt%)	A (Å)	B (Å)	C (Å)	c/a	Volume (Å^3^)
*x* = 0.005	14.655	*Amm*2 (86.19)	3.9511	5.6432	5.6761	-	126.560
		*P*4*mm* (13.81)	3.9894	3.9894	4.0173	1.0070	63.939
*x* = 0.015	13.918	*Amm*2 (97.37)	3.9555	5.6348	5.6557	-	126.057
		*P*4*mm* (2.43)	4.0199	4.0199	4.0491	1.0072	65.435
		$\mathrm{Pm}\bar{3}$*m* (0.20)	3.9361	3.9361	3.9361	-	60.983
*x* = 0.025	13.902	*P*4*mm* (98.85)	4.0074	4.0074	4.0284	1.0052	64.698
		*Amm*2 (0.82)	3.9671	5.7171	5.7257	-	129.860
		$\mathrm{Pm}\bar{3}$*m* (0.33)	3.9035	3.9035	3.9035	-	59.477
*x* = 0.035	13.589	*P*4*mm* (97.91)	3.9691	3.9691	3.9943	1.0063	62.926
		$\mathrm{Pm}\bar{3}$*m* (2.09)	3.9462	3.9462	3.9462	-	61.454

**Table 2:** Summary of dielectric and relaxor-related parameters for KNN-*x*La ceramics.

Composition (x)	*ε*_r_ @RT	tan*δ* @RT	*T*_c_ or *T*_m_ (°C)	*ε*_m_ (@*T*_m_)	*γ*	*C* (°C)
0.005	528	0.064	369	4306	1.39	8.99 × 10^5^
0.015	1316	0.029	366	1892	1.65	3.37 × 10^6^
0.025	1243	0.022	179	1817	1.71	1.72 × 10^7^
0.030	1356	0.024	170	2009	1.79	2.00 × 10^7^
0.035	1184	0.021	166	1609	1.79	1.00 × 10^7^
0.040	984	0.018	163	1287	1.94	3.00 × 10^7^

**Table 3:** Comparison of optical and piezoelectric properties of various KNN-based transparent ceramics.

Material System	Sintering Method	*T* (%) @ Visible	*T* (%) @ NIR	*d*_33_ (pC/N)	Ref.
KNLNB–Sr/Ba	hot-pressing	~60%	-	151	[17]
KNN-Eu	Solid-state	~60%	~75%	~15	[32]
KNN-Sm	Solid-state	63%	~75%	158	[27]
KNN-ErBiO_3_	Solid-state	~45%	~60%	75	[19]
KNN-Sr(Mg_1/3_Nb_2/3_)O_3_	Solid-state	~50%	~60%	92	[8]
KNN-Ca(Zn_1/3_Nb_2/3_)O_3_	Solid-state	~55%	~70%	108	[9]
KNN-La	Solid-state	~57%	~72%	92	This work

## Data Availability

The original contributions presented in this study are included in the article. Further inquiries can be directed to the corresponding author.
